# Depressive Symptoms are the Main Predictor for Subjective Sleep Quality in Patients with Mild Cognitive Impairment—A Controlled Study

**DOI:** 10.1371/journal.pone.0128139

**Published:** 2015-06-19

**Authors:** Stefan Seidel, Peter Dal-Bianco, Eleonore Pablik, Nina Müller, Claudia Schadenhofer, Claus Lamm, Gerhard Klösch, Doris Moser, Stefanie Klug, Gisela Pusswald, Eduard Auff, Johann Lehrner

**Affiliations:** 1 Department of Neurology, Medical University of Vienna, Vienna, Austria; 2 Department of Medical Statistics, Medical University of Vienna, Vienna, Austria; 3 Faculty of Psychology, University of Vienna, Vienna, Austria; University Of São Paulo, BRAZIL

## Abstract

**Objective:**

Controlled data on predictors of subjective sleep quality in patients with memory complaints are sparse. To improve the amount of comprehensive data on this topic, we assessed factors associated with subjective sleep quality in patients from our memory clinic and healthy individuals.

**Methods:**

Between February 2012 and August 2014 patients with mild cognitive impairment (MCI) and subjective cognitive decline (SCD) from our memory clinic and healthy controls were recruited. Apart from a detailed neuropsychological assessment, the subjective sleep quality, daytime sleepiness and depressive symptoms were assessed using the Pittsburgh Sleep Quality Index (PSQI), the Epworth Sleepiness Scale (ESS) and the Beck Depression Inventory (BDI-II).

**Results:**

One hundred fifty eight consecutive patients (132 (84%) MCI patients and 26 (16%) SCD patients) and 75 healthy controls were included in the study. Pairwise comparison of PSQI scores showed that non-amnestic MCI (naMCI) patients (5.4±3.5) had significantly higher PSQI scores than controls (4.3±2.8, p = .003) Pairwise comparison of PSQI subscores showed that naMCI patients (1.1±0.4) had significantly more “sleep disturbances” than controls (0.9±0.5, p=.003). Amnestic MCI (aMCI) (0.8±1.2, p = .006) and naMCI patients (0.7±1.2, p = .002) used “sleep medication” significantly more often than controls (0.1±0.6) Both, aMCI (11.5±8.6, p<.001) and naMCI (11.5±8.6, p<.001) patients showed significantly higher BDI-II scores than healthy controls (6.1±5.3). Linear regression analysis showed that the subjective sleep quality was predicted by depressive symptoms in aMCI (p<.0001) and naMCI (p<.0001) patients as well as controls (p<.0001). This means, that more depressive symptoms worsened subjective sleep quality. In aMCI patients we also found a significant interaction between depressive symptoms and global cognitive function (p = .002)

**Discussion:**

Depressive symptoms were the main predictor of subjective sleep quality in MCI patients and controls, but not in SCD patients. Better global cognitive function ameliorated the negative effect of depressive symptoms on the subjective sleep quality in aMCI patients.

## Introduction

Based on a growing pile of scientific evidence we know that cognitive deficits, mood disorders and non-restorative sleep are linked somehow[1]. Depending on the definition of mild cognitive impairment (MCI) between 14% and 63% of MCI patients report non-restorative sleep[2,3]. Cross-sectional studies have reported an inverted u-shaped relationship between sleep quality and cognitive status, with moderately demented individuals showing more impaired sleep than individuals who are in the early and advanced stages of disease[4,5]. Naismith and colleagues elegantly demonstrated that sleep-wake disturbances were linked to poor neuropsychological functioning, even after controlling for depression and apnea severity[6]. Depressive symptoms, cognition, antidepressant usage, alcohol consumption, age and education have been identified as significant predictors of self-reported sleep quality in MCI patients[3].

Subjective cognitive decline (SCD[7]) in the elderly has rather been linked to depressive symptoms than to a deterioration of objective cognitive performance[8]. SCD patients also suffer from insomnia more frequently than healthy controls[9]. A longitudinal study in older men found that disruption of the circadian rest-activity rhythm contributed to the worsening of depressive symptoms[10].

To the best of our knowledge, no other study has previously attempted to investigate independent predictors of the subjective sleep quality in patients with SCD and MCI using a controlled study design. Therefore, we performed a cross-sectional study on patients from our outpatient department for memory disorders and healthy controls and assessed potential predictors of their subjective sleep quality.

## Materials and Methods

Between February 2012 and May 2014 all consecutive patients at the outpatient department for memory disorders, who had either been referred by a neurologist or visited for a follow-up exam, underwent a semistructured interview covering general medical history, a neurological exam and a detailed neuropsychological test[11]. The study protocol was in accordance with the Helsinki Declaration and approved by the Ethical Committee of the Medical University of Vienna. Written consent was obtained from each participant.

Inclusion and exclusion criteria were similar to a recently published study by our group. Patients were excluded from the study if any of the following conditions applied: (i) evidence of stroke as determined by neuroradiological and clinical examination, (ii) history of a traumatic head injury, (iii) current psychiatric diagnosis according to ICD-10[12], however, patients with (sub-) depressive symptoms were included because (sub-) depressive symptoms often occur in elderly patients, (iv) any medical condition that leads to severe cognitive deterioration including renal, respiratory, cardiac, and hepatic disease, and (v) diagnosis of dementia according to DSM IV[13].

After the completion of the evaluation, the cognitive status of MCI subtypes was determined according to the Petersen criteria[14], and the cut-off score used was 1.5 standard deviations below age and education corrected norms using a normative sample of cognitively healthy controls. For this purpose, the flexible GAMLSS (Generalized Additive Models for Location, Scale and Shape) model class was used[15,16]. The minimum mode of MCI classification was used and patients were divided into three groups of patients based on cognitive features as follows: subjective cognitive impairment (SCD) patients (all mean z-scores of each neuropsychological test were greater than −1.5 SD), amnestic MCI (aMCI) (at least one z-score of the memory test was below −1.5 SD) and non-amnestic MCI (naMCI) patients (one z-scores of at least one domain other than memory domain was below −1.5 SD), respectively. SCD was defined according to the research criteria published by Jessen et al.[7]: (1) self-experienced persistent decline in cognitive capacity in comparison with a previously normal status and unrelated to an acute event and (2) normal age-, gender-, and education-adjusted performance on standardized cognitive tests, which are used to classify mild cognitive impairment (MCI) or prodromal Alzheimer’s dementia (AD). For the assessment of subjective memory complaint (SMC), the Forgetfulness Assessment Inventory (FAI) scale was used[11, 17].

Great care was taken enrolling a sufficient number of cognitively healthy control subjects living independently at home. They underwent a rigorous screening evaluation using a standardized clinical interview and cognitive screening. Imaging procedures, neurological examination, standard laboratory blood tests, and informant reports were not included in the evaluation. They were assessed as being in good health. Criteria for healthy function were identified as being similar to those in the Mayo research studies[18]: (i) no active neurological or psychiatric disease, (ii) no psychotropic medications, and (iii) the subjects may have medical disorders but neither they nor their treatment compromises cognitive function. Cognitive status was given special attention and cognitively healthy control subjects were screened for intact cognition. They were required to have a score ≥27 on the Mini-Mental State Examination[19].

Subjective sleep quality was assessed using the German version of the Pittsburgh Sleep Quality Index (PSQI)[20] a questionnaire that measures sleep quality over the previous month using 7 subscales measuring different components of sleep: subjective sleep quality, sleep latency, sleep duration, habitual sleep efficiency, sleep disturbances, use of sleep medication, and daytime dysfunction. Each component is reflected by a score ranging from 0 to 3, whereby 3 indicates the worse sleep quality. Good sleepers were defined as individuals with a PSQI score <5 and poor sleepers as individuals with a PSQI score ≥5.

The Epworth Sleepiness Scale (ESS)[21] is also a self-rating instrument to evaluate the tendency to doze off during daytime. It consists of eight items concerning everyday situations. Reponses to each item are ranked from 0 to 3 according to the probability for dozing off during a task (0 = never, 1 = low probability, 2 = moderate probability, 3 = high probability). A score ≥10 indicates excessive daytime sleepiness.

The Beck Depression Inventory (BDI-II), a 21-item instrument to detect depressive symptoms in adults was used. It asks about how often one felt certain ways within the past two weeks, rated on a four-point scale. A score >10 is indicative of a clinically relevant depressive symptoms[22].

### Statistical analysis

Demographic variables are described by means and standard deviations. In order to compare dependent variables between groups and subgroups, t-tests, Chi square tests and one-way ANOVAs have been computed. Uncorrected p-values are given. Post-hoc pairwise comparisons have been adjusted using Tukey’s HSD. Spearman’s correlation analysis was used to analyze the relationship between the subjective sleep quality (PSQI score) and demographic, clinical parameters and neuropsychological scores.

As possible explanatory variables for the subjective sleep quality (i.e. PSQI score) we used the years of education, BDI-II score, MMSE score, diagnosis (aMCI, naMCI, SCD and control) and ESS score. Due to their skewed distribution the variables PSQI score and BDI-II score were transformed: square root transformation was chosen instead of logarithmic transformation to avoid problems with zero values. Model selection was done by stepwise forward selection according to the best AIC. Interactions were only tested if at least one of the interacting variables was already accepted in the model.

For the logistic regression analysis the widely accepted cut-off for the PSQI (i.e. 5 points)[20] was employed and patients and controls dichotomized into good sleepers with a PSQI score < 5 vs. poor sleeper with a PSQI score ≥ 5. Explanatory variables and type of model selection was identical to the linear regression model.

Linear regression analysis was used to test the factors age, sex, years of education, ESS, BDI and PSQI scores as predictors for the cognitive performance (MMSE score).

P-values<0.05 were considered to be statistically significant. All computations were performed using SPSS statistical software, Version 20.0, except GAMLSS estimation, which was done using R 2.11.1.

## Results

One hundred fifty eight consecutive patients complaining about memory problems who came to the memory outpatient clinic for assessment of a possible cognitive disorder fulfilled the inclusion criteria and were included in the study. Patients were either referred by physicians or were self-referrals.

Fifty seven (37%) patients were classified as aMCI, 75 (47%) as naMCI, and 26 (16%) as SCD as described above. We also included 75 healthy controls in this study. Demographic and clinical characteristics of patients and controls can be found in Table 1.

**Table 1:** Demographic and clinical characteristics of patients and controls.

	aMCI	naMCI	SCD	Controls	p-value
*Sex (f*:*m)*	28:29	43:32	16:10	29:46	.524
*Age (mean±SD)*	67.7±9.1	69.8±7.2	65.7±8.8	68.4±11.8	.311
*Years of education (mean±SD)*	12.8*±4*.*0*	12.2±4.0	12.9*±3*.*6*	13.0*±3*.*9*	.594
*Regular sleep medication (n (%))*	16 (21)	13 (23)	7 (27)	0 (0)	**< .001**
*MMSE score (mean±SD)*	27.3±3.2	28.3±1.5	29.1±1.0	28.5±1.1	**< .001**
*BDI-II score (mean±SD)*	11.5±8.6	11.5±8.6	9.0±6.4	5.0±5.3	**< .001**
*ESS score (mean±SD)*	6.6±4.2	8.2±3.8	8.0±4.0	6.9±3.2	.062
*Good sleepers (PSQI<5*, *n (%))*	31 (41)	28 (49)	12 (46)	48 (64)	**.042**
*PSQI score (mean±SD)*	6.3±4.0	5.4±3.5	6.0±3.9	4.3±2.8	**.005**
*PSQI subscores*					
Subjective sleep quality	1.0±0.7	0.8±0.7	1.1±0.8	0.8±0.5	.066
Sleep latency	1.0±0.9	0.8±0.9	0.5±1.0	0.8±0.8	.094
Sleep duration	0.3±0.7	0.6±1.0	0.8±1.1	0.4±0.7	**.005**
Habitual sleep efficiency	0.8±1.1	0.8±1.0	0.8±1.1	0.6±1.0	.768
Sleep disturbances	1.2±0.5	1.1±0.4	1.2±0.5	0.9±0.5	**.005**
Use of sleep medication	0.8±1.2	0.7±1.2	0.7±1.1	0.1±0.6	**.001**
Daytime dysfunction	1.0±0.7	0.9±0.6	0.9±0.8	0.7±0.7	.151

aMCI = amnestic MCI patients, naMCI = non-amnestic MCI patients, SCD = subjective cognitive decline patients, MMSE = Mini Mental State Examination, PSQI = Pittsburgh Sleep Quality Index, BDI-II = Beck Depression Inventory, SD = standard deviation.

AMCI patients (27.6±1.7) showed a significantly lower MMSE score than naMCI patients (28.3±1.5, p = .02), SCD patients (29.1±1.0, p < .001) and controls (29.0±1.1, p = .002). Both, aMCI (11.5±8.6, p < .001) and naMCI (11.5±8.6, p < .001) patients showed significantly higher BDI-II scores than healthy controls (6.1±5.3) (Tables 1 and 2).

**Table 2:** Pairwise comparison of MMSE, BDI and PSQI (sub-)scores between aMCI-, naMCI-, SCD patients and controls.

MMSE	difference	lower CI	upper CI	p adj TukeyHSD
SCD vs. controls	0.597	-0.54	1.734	.527
aMCI vs. controls	-1.217	-2.095	-0.339	**.002**
naMCI vs. controls	-0.227	-1.043	0.589	.89
aMCI vs. SCD	-1.814	-2.996	-0.631	**.001**
naMCI vs. SCD	-0.824	-1.961	0.314	.242
naMCI vs. aMCI	0.99	0.112	1.868	**.02**
**BDI-II**	difference	lower CI	upper CI	p adj TukeyHSD
SCD vs. controls	2.867	-1.513	7.247	.329
aMCI vs. controls	5.323	1.941	8.705	**< .001**
naMCI vs. controls	5.32	2.177	8.463	**< .001**
aMCI vs. SCD	2.456	-2.098	7.011	.503
naMCI vs. SCD	2.453	-1.927	6.833	.47
naMCI vs. aMCI	-0.003	-3.385	3.379	1
**PSQI total score**	difference	lower CI	upper CI	p adj TukeyHSD
SCD vs. controls	1.693	-0.395	3.782	.157
aMCI vs. controls	1.097	-0.516	2.709	.295
naMCI vs. controls	2.04	0.542	3.538	**.003**
aMCI vs. SCD	-0.596	-2.768	1.575	.893
naMCI vs. SCD	0.347	-1.742	2.435	.973
naMCI vs. aMCI	0.943	-0.669	2.556	.431
**PSQI sleep duration**	difference	lower CI	upper CI	p adj TukeyHSD
SCD vs. controls	0.44	-0.081	0.961	.13
aMCI vs. controls	-0.067	-0.463	0.33	.972
naMCI vs. controls	0.24	-0.128	0.608	.334
aMCI vs. SCD	-0.507	-1.048	0.035	.076
naMCI vs. SCD	-0.2	-0.721	0.321	.753
naMCI vs. aMCI	0.307	-0.09	0.703	.19
**PSQI sleep disturbances**	difference	lower CI	upper CI	p adj TukeyHSD
SCD vs. controls	0.221	-0.064	0.507	.189
aMCI vs. controls	0.12	-0.101	0.341	.495
naMCI vs. controls	0.281	0.076	0.486	**.003**
aMCI vs. SCD	-0.101	-0.398	0.195	.814
naMCI vs. SCD	0.059	-0.226	0.345	.949
naMCI vs. aMCI	0.161	-0.06	0.381	.236
**PSQI use of sleep medication**	difference	lower CI	upper CI	p adj TukeyHSD
SCD vs. controls	0.519	-0.091	1.128	.126
aMCI vs. controls	0.602	0.131	1.073	**.006**
naMCI vs. controls	0.625	0.187	1.063	**.002**
aMCI vs. SCD	0.083	-0.55	0.716	.986
naMCI vs. SCD	0.106	-0.502	0.715	.969
naMCI vs. aMCI	0.023	-0.447	0.493	.999

aMCI = amnestic MCI, naMCI = non-amnestic MCI, SCD = subjective cognitive decline, MMSE = Mini Mental State Examination, BDI-II = Beck Depression Inventory, PSQI = Pittsburgh Sleep Quality Index, p adj Tukey HSD = adjusted p-values for post-hoc Tukey’s HSD, CI = 95% confidence interval.

The MMSE score was inversely correlated with age (p < .001) and directly correlated with the years of education (p < .001). Linear regression analysis showed that the age (p < .001) and years of education (p < .001) were significant predictors for the MMSE score, meaning that higher age predicated a lower MMSE score and a higher number of years of education predicated a higher MMSE score.

### Subjective sleep quality, sleep medication and daytime sleepiness

Thirty one (41%) aMCI and 28 (49%) naMCI patients), 12 (46%) SCD patients and 48 (64%) healthy controls were good sleepers (i.e. had PSQI scores < 5 points).

Table 1 shows the PSQI (sub-)scores and ESS scores for all patient groups and controls. Univariate ANOVA showed significant differences of the PSQI scores (p = .005) and the PSQI subscores “sleep duration” (p = .005), “sleep disturbances” (p = .005) and “use of sleep medication” (p = .001) (Table 1). Pairwise comparison of PSQI scores showed that naMCI patients (5.4±3.5) had significantly higher PSQI scores than controls (4.3±2.8, p = .003) (Table 2). Pairwise comparison of PSQI subscores showed that naMCI patients (1.1±0.4) had significantly more “sleep disturbances” than controls (0.9±0.5, p = .003). AMCI (0.8±1.2, p = .006) and naMCI patients (0.7±1.2, p = .002) used “sleep medication” significantly more often than controls (0.1±0.6) (Table 2).

Thirteen (23%) aMCI and 17 (23%) naMCI patients and 7 (27%) SCD patients, and none of the healthy controls took sleep medication on a daily basis (p < .001). Antidepressants (i.e. trazodone or mirtazapine) were used by 6 (46%) aMCI, 12 (70%) naMCI and 6 (85%) SCD patients. Benzodiazepines (i.e. triazolam or diazepam) or a benzodiazepine-receptor-agonist (i.e. zolpidem) were used by 5 (38%) aMCI, 3 (18%) naMCI and none of the SCD patients. The antipsychotic prothipendyl was used by 2 (15%) aMCI, 2 (12%) naMCI and 1 (17%) SCD patients.

The level of daytime sleepiness (ESS scores) did not differ significantly between patients and controls (Table 1).

**Fig 1:**
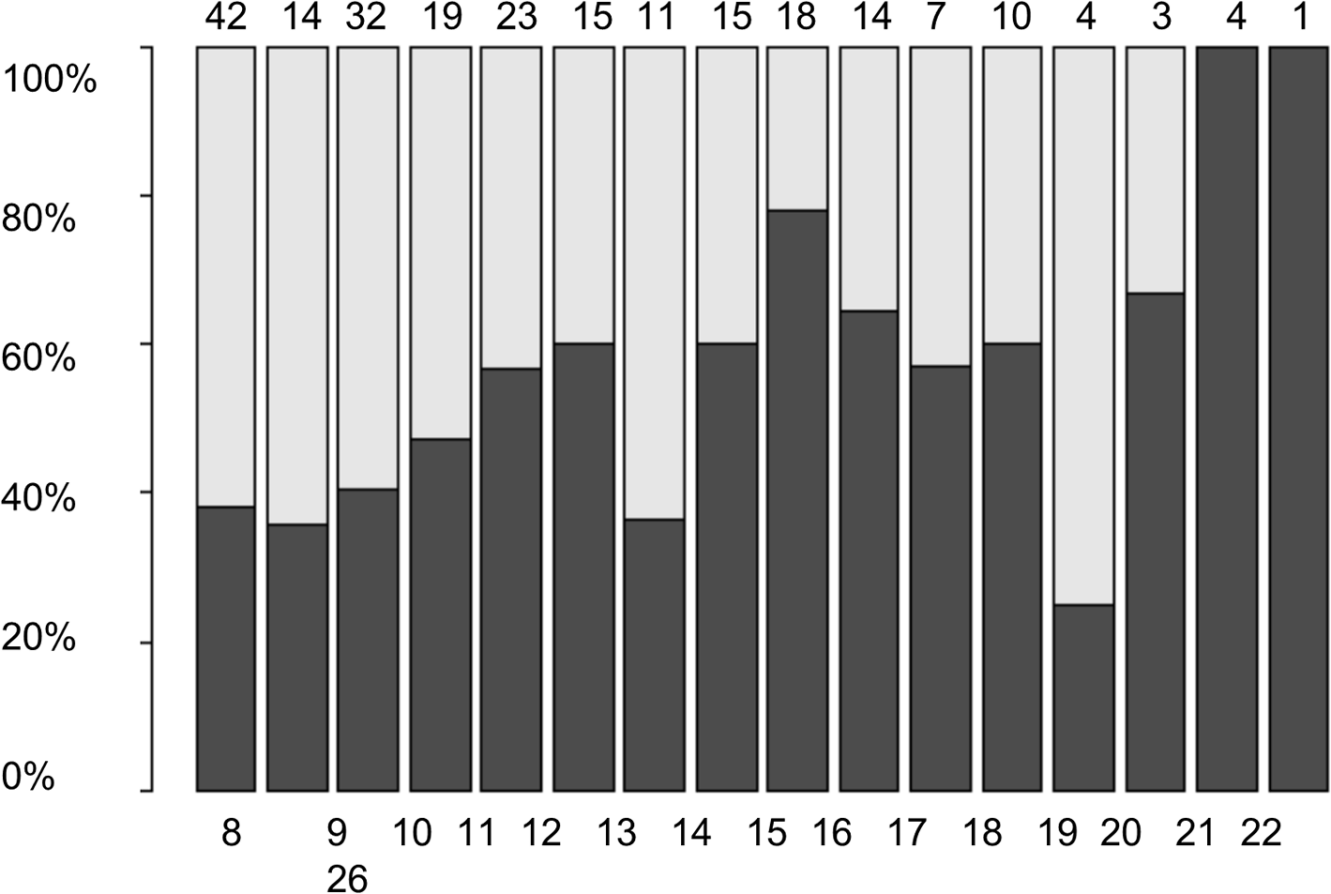
Relative frequency (in percent) of good (black) and bad (white) sleepers in each group of patients with the same number of years of education. The number of patients is shown above the bars and the number of years of education is shown below the bars.

**Fig 2:**
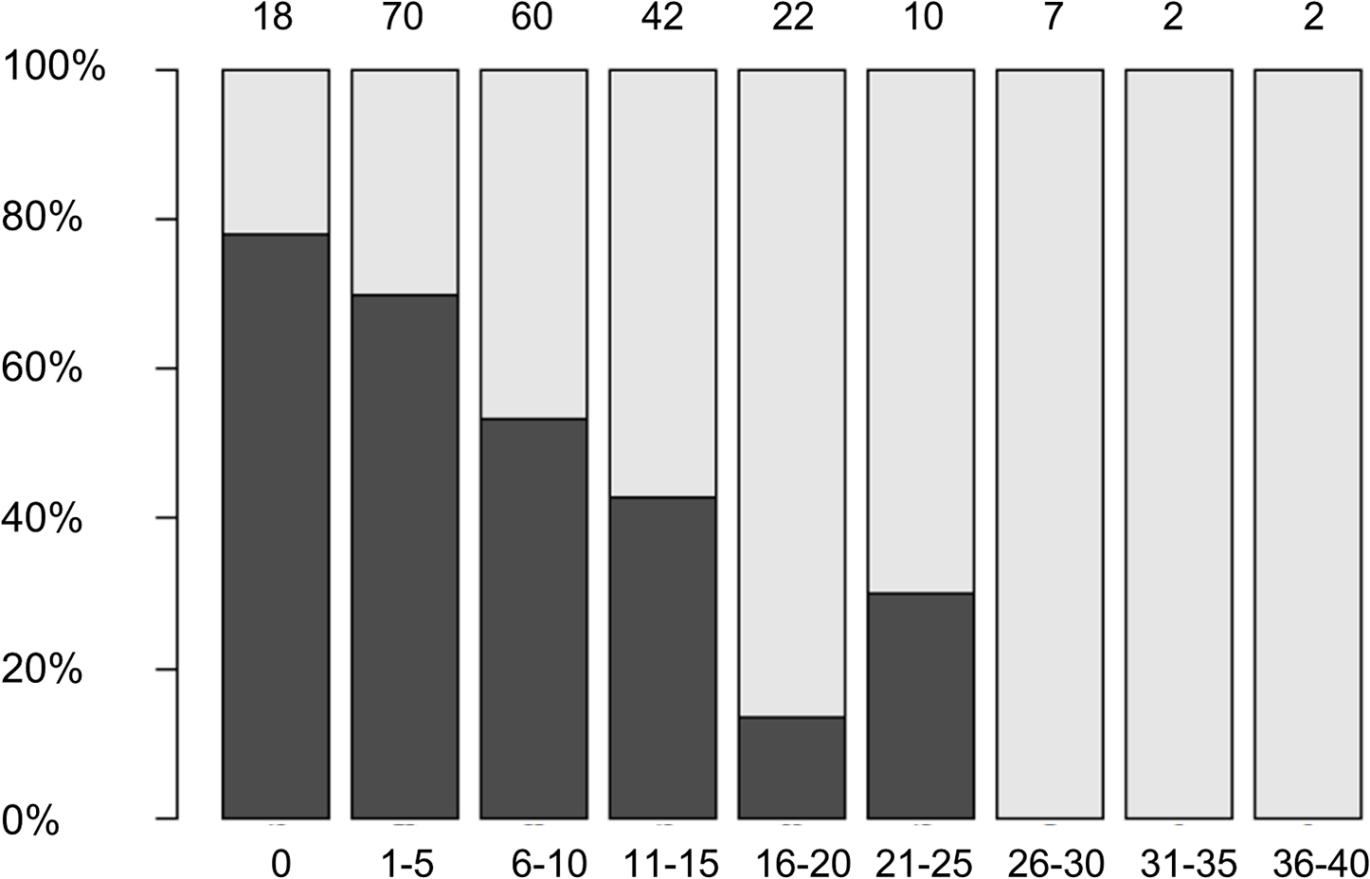
Relative frequency of good (black) and bad (white) sleepers in each group of patients with similar BDI scores. The number of patients is shown above the bars and the range of BDI score for each subgroup is shown below the bars.

The subjective sleep quality (PSQI score) was inversely correlated with the years of education (p < .001) and directly correlated with depressive symptoms (BDI-II score) (p < .001), meaning the more years of education the better the sleep quality (i.e. lower PSQI scores) and the more depressive symptoms the worse the sleep quality (i.e. higher PSQI scores). Figs 1 and 2 show the relative frequencies of good vs. poor sleepers with respect to the years of education and depressive symptoms (BDI-II scores).

The best linear regression model for the metric variable PSQI score is shown in Table 3. This model showed that depressive symptoms (BDI-II score) (p < .0001) and, to a lesser extent, the years of education (p = .030) significantly predicted the subjective sleep quality (PSQI score). We also found a significant interaction between depressive symptoms (BDI-II score) and global cognitive function (MMSE score) (p < .001), meaning that the direct correlation between subjective sleep quality (PSQI score) and depressive symptoms (BDI-II score) was alleviated by better global cognitive function (increased MMSE scores) (Table 3).

**Table 3:** Best linear regression model for PSQI score among the whole sample.

	estimate	p-value (Type IIISSQ)
years of education	-0.0249	**.030**
BDI-II score	0.2875	**< .00001**
Interaction BDI-II score:MMSE	-0.1356	**< .001**

MMSE = Mini Mental State Examination, BDI-II = Beck Depression Inventory

The best logistic regression model for the dichotomized PSQI score (cut-off 5 points) is shown in Table 4. This model showed that depressive symptoms (BDI-II score) (p < .0001) and to a lesser extent the years of education (p = .030) significantly predicted the sleep status. We did not observe a significant interaction between the BDI-II and the MMSE score in this model.

**Table 4:** Best logistic regression model for the sleep status among the whole sample.

	estimate	p-value (Type IIISSQ)
Years of education	-0.0249	**.030**
BDI-II score	0.2875	**< .00001**

BDI-II = Beck Depression Inventory

We re-ran the calculation of the two final models for each subgroup (aMCI, naMCI, SCD and controls) separately to check whether the observed relationships in the full model were mainly driven by one or two particular subgroups (Tables 5 and 6). These calculations showed that the subjective sleep quality (PSQI score) was significantly predicted by the depressive symptoms in aMCI (p < .0001) and naMCI (p < .0001) patients as well as controls (p < .0001). In aMCI patients we also found a significant interaction between depressive symptoms (BDI-II score) and global cognitive function (MMSE score) (p = .002) (Table 5) as we did for the whole sample (Table 3). The sleep status was significantly predicted by the depressive symptoms in aMCI (p < .001) and naMCI (p < .001) patients as well as controls (p = .042). Only in naMCI patients the sleep status was also significantly predicted by the years of education (p = .027) (Table 6).

**Table 5:** Best linear regression models for PSQI score for each subgroup.

**Controls**				
Coefficients:	Estimate	Std. Error	t value	Pr(>|t|)
(Intercept)	1.384501	0.322584	4.292	**< .00001**
Year of education	-0.004227	0.020352	-0.208	.836
Sqrt (BDI-II score)	0.278614	0.062088	4.487	**< .00001**
Interaction BDI-II:MMSE	-0.095893	0.084700	-1.132	.261
**SCD patients**				
Coefficients:	Estimate	Std. Error	t value	Pr(>|t|)
(Intercept)	2.88251	0.70178	4.107	**< .001**
Year of education	-0.06943	0.04478	-1.550	.136
Sqrt (BDI-II score)	0.12910	0.11829	1.091	.287
Interaction BDI-II:MMSE	0.01274	0.18223	0.070	.945
**aMCI patients**				
Coefficients:	Estimate	Std. Error	t value	Pr(>|t|)
(Intercept)	1.41045	0.34221	4.122	**< .001**
Year of education	-0.01293	0.01952	-0.663	.510
Sqrt (BDI-II score)	0.31828	0.05570	5.715	**< .0001**
Interaction BDI-II:MMSE	-0.17665	0.05519	-3.201	**.002**
**naMCI patients**				
Coefficients:	Estimate	Std. Error	t value	Pr(>|t|)
(Intercept)	1.94479	0.34317	5.667	**< .0001**
Year of education	-0.04072	0.02127	-1.914	.060
Sqrt (BDI-II score)	0.29777	0.06628	4.493	**< .0001**
Interaction BDI-II:MMSE	-0.07582	0.08249	-0.919	.361

aMCI = amnestic MCI, naMCI = non-amnestic MCI, SCD = subjective cognitive decline, MMSE = Mini Mental State Examination, BDI-II = Beck Depression Inventory

**Table 6:** Best logistic regression models for the sleep status for each subgroup.

**Controls**				
Coefficients:	Estimate	Std. Error	t value	Pr(>|z|)
(Intercept)	-0.61328	1.00545	-0.610	.542
BDI-II score	0.09589	0.04854	1.976	**.048**
Years of education	-0.04501	0.06731	-0.669	.504
**SCD patients**				
Coefficients:	Estimate	Std. Error	t value	Pr(>|z|)
(Intercept)	0.92046	1.71721	0.536	.592
BDI-II score	0.07247	0.06951	1.043	.297
Years of education	-0.11323	0.11969	-0.946	.344
**aMCI patients**				
Coefficients:	Estimate	Std. Error	t value	Pr(>|z|)
(Intercept)	-0.41204	1.21259	-0.340	.734
BDI-II score	0.17437	0.05215	3.344	**< .001**
Years of education	-0.11208	0.08540	-1.313	.189
**naMCI patients**				
Coefficients:	Estimate	Std. Error	t value	Pr(>|z|)
(Intercept)	0.66517	0.89650	0.742	.458
BDI-II score	0.15156	0.04615	3.284	**.001**
Years of education	-0.15054	0.06794	-2.216	**.027**

aMCI = amnestic MCI, naMCI = non-amnestic MCI, SCD = subjective cognitive decline, MMSE = Mini Mental State Examination, BDI-II = Beck Depression Inventory

### Subjective sleep quality and neuropsychological test scores

We also investigated the relationship between the subjective sleep quality (PSQI score) and neuropsychological test scores in the whole sample. We found weak but significant positive correlations (i.e. the better the neuropsychological test score the worse the subjective sleep quality, represented by a higher PSQI score) between the PSQI score and items from domain 1 “attention”, i.e. AKT time (p = .008), Symbols counting (C.I.) (p = .004), domain 3 “executive function–interference”, i.e. interference (C.I.) time (p = .037), and domain 6 “executive function–planning and nonverbal fluency”, i.e. Planning Maze Test–NAI time (p = .036) (Table 7). We found weak but significant negative correlations (i.e. the better the neuropsychological test score the better the subjective sleep quality, represented by a lower PSQI score) between the PSQI score and items from domain 1 “attention”, i.e. AKT total/time (p = .003) and Digital-Symbol Test (WAIS-R) (p = .006), domain 4 “language”, i.e. Boston Naming Test (mBNT) (p = .012) and domain 6 “executive function–planning and nonverbal fluency”, i.e. Planning Maze Test–NAI total/time (p = .020) and Nonverbal Fluency Five-Point Test–total correct (p = .024) (Table 7). Due to the small subgroup sizes we did not perform these calculations for each subgroup.

**Table 7:** Spearman correlation coefficients for the whole sample between PSQI total scores and neuropsychological subdomains.

Neuropsychological test	Spearman’ cc	p-Value
**Domain 1 attention**		
AKT time	.173	**.008**
AKT total/time	-.193	**.003**
Trail-Making Test–TMTB	.074	.261
Digit-Symbol Test (WAIS-R)	-.123	**.006**
TMTB–TMTA difference	.085	.196
Symbols counting (C.I.)	.186	**.004**
**Domain 2 executive function–phonemic verbal fluency**		
Phonematic verbal fluency PWT total words	-.025	.709
Phonematic verbal fluency PWT l-words	.004	.947
Phonematic verbal fluency PWT f-words	-.088	.181
Phonematic verbal fluency PWT b-words	.022	.735
**Domain 3 executive function—interference**		
Stroop color words	.051	.438
Stroop total/time	-.099	.135
Interference (C.I.) time	.137	**.037**
Interference (C.I.) total/time	-.119	.070
Stroop color words–colors	.008	.902
Stroop colors	.143	.029
**Domain 4 language**		
Semantic verbal fluency SWT total words	-.101	.125
Semantic verbal fluency SWT supermarket items	-.053	.419
Semantic verbal fluency SWT animals	-.104	.112
Semantic verbal fluency SWT tools	-.104	.115
Boston Naming Test (mBNT)	-.165	**.012**
**Domain 5 memory**		
Verbal memory total recall (VSRT)	.061	.354
Verbal memory immediate recall (VSRT)	.079	.232
Verbal memory delayed recall (VSRT)	.050	.449
Verbal memory recognition (VSRT)	.117	.076
**Domain 6 executive function–planning and nonverbal fluency**		
Planning Maze Test–NAI time	.138	**.036**
Planning Maze Test–NAI total/time	-.154	**.020**
Nonverbal Fluency Five-Point Test–total correct	-.148	**.024**
Trail-Making Test–TMTA	-.002	.978
Nonverbal Fluency Five-Point Test–perseverations	.086	.189

Cc = correlation coefficient; AKT = Alters-Konzentrations-Test; WAIS-R = Wechsler Adult Intelligence Scale—Revised; TMTA = Trail Making Test Version A; TMTB = Trail Making Test Version B; NAI = Nürnberger Alters Inventar; C.I. = Cerebral Insufficiency Test; VSRT = Verbal Selective Reminding Test; mBNT = modified Boston Naming Test.

## Discussion

This is the first controlled study investigating predictors of subjective sleep quality which included patients with objective (MCI) and subjective memory impairment (SCD). Depressive symptoms were the main predictor of subjective sleep quality in MCI patients and controls. Educational attainment predicted the subjective sleep quality only in naMCI patients. Increased global cognitive function appeared to mitigate the relationship between depressive symptoms and subjective sleep quality in aMCI patients.

Similar to a previous study[23,24] we found a higher frequency of depressive symptoms in aMCI and naMCI patients compared to controls. Ausén et al. failed to detect differences of depressive symptoms between patients with subjective cognitive impairment, MCI patients and controls[23]. Interestingly, regarding their subjective sleep quality or depressive symptoms our SCD patients did not differ significantly from MCI patients or controls either. However, the number of SCD patients using sleep medication, predominantly antidepressants, on a regular basis was comparable to aMCI and naMCI patients. A recent study showed that the use of sleep medication was independently associated with depression in older adults[25]. The fact that these SCD patients were taking sleep medication may already have improved their subjective sleep quality and thus, their (subclinical) depression. The lack of differences between SCD and MCI patients may also be due to a “contamination” of SCD by early MCI since we know that neurodegeneration evolves continually[26,27]. Moreover, we cannot rule out lack of statistical power due to the small subgroup sizes.

Previous research on predictors of subjective sleep quality in patients with memory impairment has yielded conflicting data[28]. McKinnon et al. [3]reported that depressive symptoms explained the largest portion of variance in PSQI (sub-)scores, which is line with our findings. While other authors[3] found an array of variables to predict the subjective sleep quality in patients with memory impairment, we could only identify education the other significant predictor. This sociodemographic variable is a surrogate of general health-related factors (i.e. body mass index, physical activity, smoking status, alcohol consumption etc.). The positive effect of education on sleep quality has been illustrated previously in the general population[29]. However, recently, it was shown that medical burden, physical exercise and body mass index were not associated with subjective sleep quality in MCI patients[3].

We confirm the findings of other studies comparing the subjective sleep quality of MCI subtypes [30], which did not detect any differences between aMCI and naMCI patients. Notably, our naMCI patients had significantly more trouble maintaining sleep than controls. Decreased maintenance of sleep has previously been shown to differentiate naMCI from aMCI who reported difficulties initiating sleep and early morning awakenings[31].

Our finding that increased global cognitive function (MMSE score) mitigated the negative effect of depressive symptoms on the subjective sleep quality in aMCI patients leads us into the discussion of the relationship between neurodegeneration, mood and sleep-wake disorders. Due to the small subgroup sizes we may not present consistent data on neuropsychological subdomains, but we like to speculate on the neuroanatomical and functional relationships. To us, the hippocampus with its widespread connections lies at the core of these interwined conditions. During non-REM sleep it plays an essential role in the consolidation of declarative memories[32]. Sleep restriction causes disrupted functional connectivity in neural circuits involving networks responsible for emotional and executive processing[33]. Depression is not only associated with decreased hippocampal volume[34] but also impaired corticohippocampal connectivity[35]. Grey matter volume within the above mentioned networks has been shown to be decreased in MCI patients[36]. Education serves as one of the positive modulators of hippocampal volume[37].

Excessive daytime sleepiness (EDS) has been associated with an increased risk for cognitive decline[38]. Ferman et al. showed that MCI patients with EDS were more likely to develop dementia with Lewy bodies[39]. Daytime sleepiness is also associated with sleep apnea[40]. A controlled study failed to detect an association between sleep disordered breathing (SDB) and MCI[41]. We did not observe significant differences of EDS between patients and controls. One has to keep in mind that self-report scales like the ESS may not be sensitive enough to detect more subtle changes of our sleep-wake behavior. Disruption of functional connectivity in the resting state-network has recently been associated with daytime sleepiness[42].

Our study is limited by the lack of objective sleep parameters (actigraphy and/or polysomnography) and therefore we cannot fully rule out sleep-disordered-breathing or periodic limb movements during sleep in our sample. The PSQI relies entirely on self-report, but the problem of sleep misperception with an overestimation of sleep onset latency by MCI patients has recently been highlighted[43]. Actigraphic assessment of circadian rhythms in our patients may have yielded additional and relevant information. A recent actigraphic study has highlighted the possible role of delayed circadian rhythms as a risk factor for cognitive decline[44]. Although a clinical assessment of depression was not conducted to establish major depression diagnoses and lifetime depressive histories, the use of the BDI, which has been reported to show high reliability and good correlation with measures of depression and anxiety[45]enabled us to properly investigate psychiatric symptoms. The hospital-based study design of our study precludes the generalizability of our findings on to population level.

To conclude, this is the first cross-sectional controlled study showing that depressive symptoms predicted the subjective sleep quality in MCI patients and healthy controls, but not in SCD patients. Educational attainment appeared to act positively on the subjective sleep quality in naMCI patients. Better global cognitive function ameliorated the negative effect of depressive symptoms on the subjective sleep quality in aMCI patients. Future longitudinal studies including objective measurements of sleep variables should clarify the relationship of mood disorders, subjective cognitive decline and sleep.
